# Autophagy: a necessary defense against extreme cadmium intoxication in a multigenerational 2D experiment

**DOI:** 10.1038/s41598-020-78316-z

**Published:** 2020-12-03

**Authors:** Agnieszka Babczyńska, Agnieszka Nowak, Alina Kafel, Bartosz Łozowski, Magdalena Rost-Roszkowska, Monika Tarnawska, Maria Augustyniak, Marta Sawadro, Agnieszka Molenda

**Affiliations:** Institute of Biology, Biotechnology and Environmental Protection, Faculty of Natural Sciences, University of Silesia in Katowice, Bankowa 9, 40-007 Katowice, Poland

**Keywords:** Autophagy, Developmental biology, Evolutionary developmental biology, Metabolism, Ecophysiology

## Abstract

Autophagy is a natural process that aims to eliminate malfunctioning cell parts, organelles or molecules under physiological conditions. It is also induced in response to infection, starvation or oxidative stress to provide energy in case of an energy deficit. The aim of this 2-dimensional study was to test if, and if so, how, this process depends on the concentration of cadmium in food (with Cd concentrations from 0 to 352 μg of Cd per g of food (dry weight)—D1 dimension) and the history of selection pressure (160 vs 20 generations of exposure to Cd—D2 dimension). For the study, the 5th instar larvae of a unique strain of the moth *Spodoptera exigua* that was selected for cadmium tolerance for 160 generations (44 μg of Cd per g of food (dry weight)), as well as 20-generation (11, 22 and 44 μg of Cd per g of food (dry weight)) and control strains, were used. Autophagy intensity was measured by means of flow cytometry and compared with life history parameters: survivability and duration of the 3rd larval stage. The highest values of autophagy markers were found in the groups exposed to the highest Cd concentration and corresponded (with a significant correlation coefficient) to an increased development duration or decreased survivorship in the respective groups. In conclusion, autophagy is probably initiated only if any other defense mechanisms, e.g., antioxidative mechanisms, are not efficient. Moreover, in individuals from pre-exposed populations, the intensity of autophagy is lower.

## Introduction

Autophagy is a highly conserved process of cellular self-destruction. Recognition of this process, which protects cells by eliminating damaged organelles or molecules, has been increasing recently, especially in relation to human health and medical sciences. Additionally, many studies have been performed using invertebrate model species. As a constitutive process in equilibrium with other programmed cellular pathways, autophagy is the basis of homeostasis and proper development, including aging processes^1–4^. Starvation is among the conditions that led to the development of autophagy as an evolutionary adaptive mechanism^5,6^. Autophagy enables the maintenance of homeostasis during winter dormancy, which is associated with food and energy deficiency, because autophagy supplies cells with nutrients^7^. Starvation is considered to cause prooxidative changes^8–11^, generating endoplasmic reticulum (ER) stress, which is recognized as a proautophagic factor^12,13^. The same mechanisms explain the proautophagic action of metals such as cadmium^14^. As a nonconstitutive process induced in the context of defense against unexpected (“not planned”) factors, autophagy may seem undesirable from a resource- and energy-saving point of view. Rabinowitz and White^15^ suggest that expenditures are connected not only with the loss (destruction) of molecules and organelles but also with the necessity of resynthesis or de novo synthesis of the destroyed components. On the other hand, the issue may be regarded as a trade-off between these energy and resource costs and the benefits associated with the neutralization of the threat or its consequences. This concept was proposed by^16^. Unfortunately, this question has not yet been investigated sufficiently, and additional reports are unavailable.

Cadmium, an autophagy inducer, has been studied mainly in human and mammalian cell lines^17,18^ but the correlation between Cd exposure and autophagy indices has also been described in invertebrates^19–21^. Chiarelli et al.^21^ supposed that there is a hierarchical system for inducing defense mechanisms and that autophagy belongs to the main but not the first response. In turn, according to Wilczek et al.^22^, the observation of autophagosomes in silk gland cells of spiders depends on the duration of Cd intoxication. The results indicated that long-term (12 months) exposure induced autophagy in the cells^22^. Considering the exposure duration and the fact that autophagy is induced later than other mechanisms for neutralizing cadmium toxicity, one may suppose that pre-exposure to cadmium may lead to lower autophagy intensity in individuals with increased Cd tolerance. This question has not yet been answered. Therefore, in this paper, an experiment involving individuals of the moth *Spodoptera exigua*, a model insect species, subjected to selection pressure towards Cd tolerance for over 160 generations was reported. This population is unique worldwide, and it originates from a laboratory strain that was reared at the Institute of Biology, Biotechnology and Environmental Protection, University of Silesia in Katowice, Poland, for over 17 years. Numerous investigations conducted by the research team revealed that during that time, this selected strain acquired specific protective mechanisms, while some life history, biochemical and physiological parameters of this strain do not differ from those of control individuals^23–29^. There is no strong evidence that this tolerance to cadmium is genetically preserved. The levels of at least some parameters connected to direct or indirect Cd neutralization seem to result from phenotypic plasticity^29^. This is related to the concentrations of stress proteins (heat shock proteins (HSP70) and metallothioneins (Mts)). Their levels in the two strains (control and Cd-selected) did not differ significantly, but in 4 new experimental strains administered food with various Cd contents, these protein concentrations reflected metal exposure^29^. This seems especially important in relation to the present study because HSP70 and Mts play an antioxidative role^30^. Therefore, an efficient antioxidative system might prevent the induction of autophagy. Additionally, although some markers changed in Cd-tolerant insects, others did not change, regardless of the duration (number of generations) of exposure. This is related to Cd accumulation in the pupae—it is constant from generation to generation. Therefore, no intake-preventing mechanisms developed, but the neutralization mechanisms appeared to be efficient. The aim of the present study was to test the following hypotheses:

H0. Autophagy initiation and intensity do not differ with either increasing cadmium concentrations or multigenerational pre-exposure to this metal, and this process is not subject to adaptive selection, similar to the case of Cd accumulation in pupae mentioned above.

H1. Autophagy is an energy-consuming process, and under exposure to toxic or harmful factors, it is initiated only if any other defense mechanisms, e.g., antioxidative processes, are not efficient; in addition, this feature does not depend on the exposure duration (counted as the number of generations of insects pre-exposed to the metal).

H2. Regardless of the fact that autophagy is initiated as the last (or one of the last) defensive process, according to the abovementioned suggestion^21^, in individuals from pre-exposed (adapted) populations, the intensity of autophagy is lower. This might be beneficial from an energy-, organelle- and molecule-saving point of view.

To connect the markers of autophagy levels to possible trade-offs between growth and development and defensive processes, two life history parameters of the insects were measured: cumulative mortality at the 3rd and 4th larval stages as well as the duration of the 3rd larval stage.

## Results

### Autophagy indices

**Figure 1:**
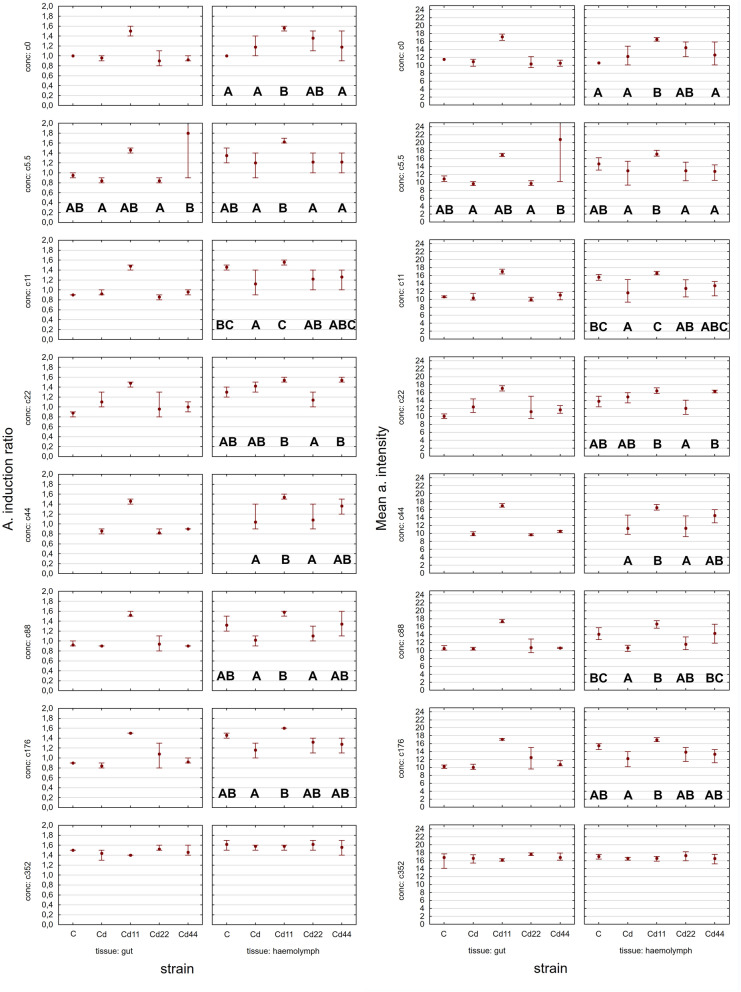
AIR (A. induction ratio) and MAI (Mean a. intensity) in the hemolymph and midgut of the 5th larval stage of the moth *S. exigua* from the 2D experiment. Means ± mean and max values. Different letters (A, B, C) denote statistically significant differences between strains (D1); ANOVA, Tukey’s test for unequal sample size, p ≤ 0.05.

The autophagy induction ratio (AIR) and mean autophagy intensity (MAI) were similar in each kind of cell (midgut cells and hemocytes). The statistical analysis of the results indicates that the levels of autophagy indices are related to both the strain (dimension D1, Tables 1, 2) and the experimental group (dimension D2, Tables 1, 2). Additionally, regarding strain-related differences, the two parameters showed the same pattern, which was specific to the kind of biological material (midgut cells or hemocytes) (Fig. 1).

**Table 1:** Analysis of variance (ANOVA/MANOVA) for AIR in *S. exigua* larvae with strain (D1) and concentration (D2) as categorical factors.

Source of variation	df	Midgut		Hemolymph	
		F	p	F	p
Strain	3	18.80025	< 0.001	58.61214	< 0.001
Concentration	6	5.31533	< 0.001	16.88001	< 0.001
Strain × concentration	27	1.50579	0.065255	3.75869	< 0.001

**Table 2:** Analysis of variance (ANOVA/MANOVA) for MAI in *S. exigua* larvae with strain (D1) and concentration (D2) as categorical factors.

Source of variation	df	Midgut		Hemolymph	
		F	p	F	p
Strain	3	18.24268	< 0.001	63.25309	< 0.001
Concentration	6	5.10479	< 0.001	17.22473	< 0.001
Strain × concentration	27	1.43174	0.092220	4.12983	< 0.001

### Hemolymph

**Figure 2:**
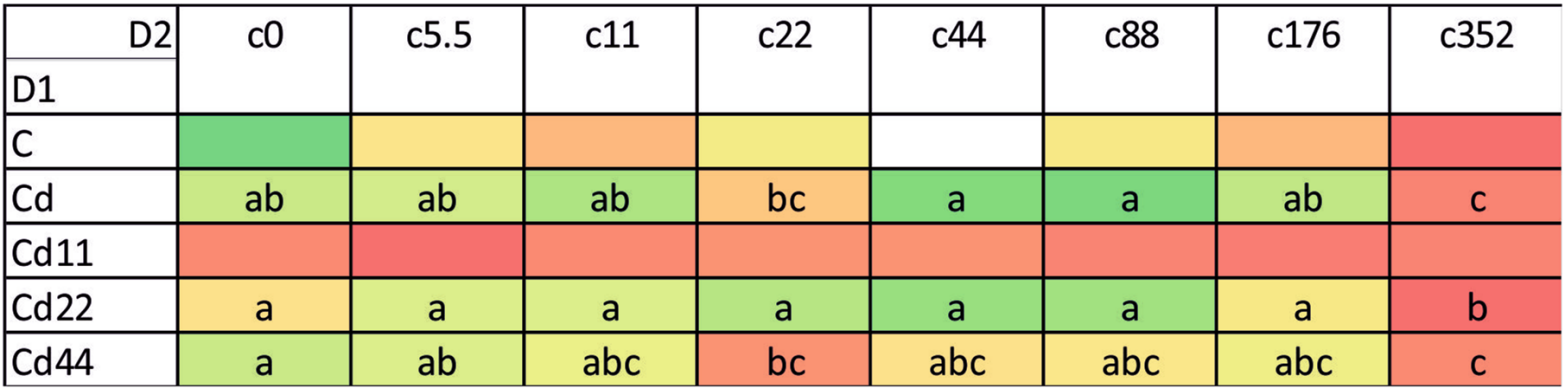
AIR in the hemolymph of the 5th larval stage of the moth *S. exigua* from the 2D experiment. D1, D2—dimensions of the experiment, according to Fig. 10. Values of the AIR are expressed according to a color scale, where dark green is the lowest and dark red is the highest. Different letters (a, b, c) denote statistically significant differences between experimental groups (D2); ANOVA, Tukey’s test for unequal sample size, p ≤ 0.05.

**Figure 3:**
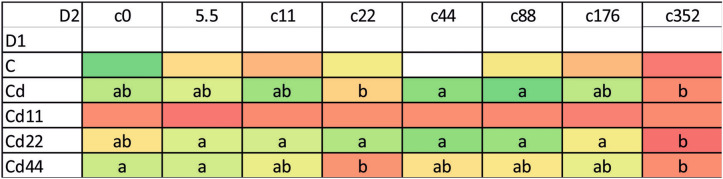
MAI in the hemolymph of the 5th larval stage of the moth *S. exigua* from the 2D experiment. D1, D2—dimensions of the experiment, according to Fig. 10. Values of the MAI are expressed according to a color scale, where dark green is the lowest and dark red is the highest. different letters (a, b) denote statistically significant differences between experimental groups (D2); ANOVA, Tukey’s test for unequal sample size, p ≤ 0.05.

**Figure 4:**
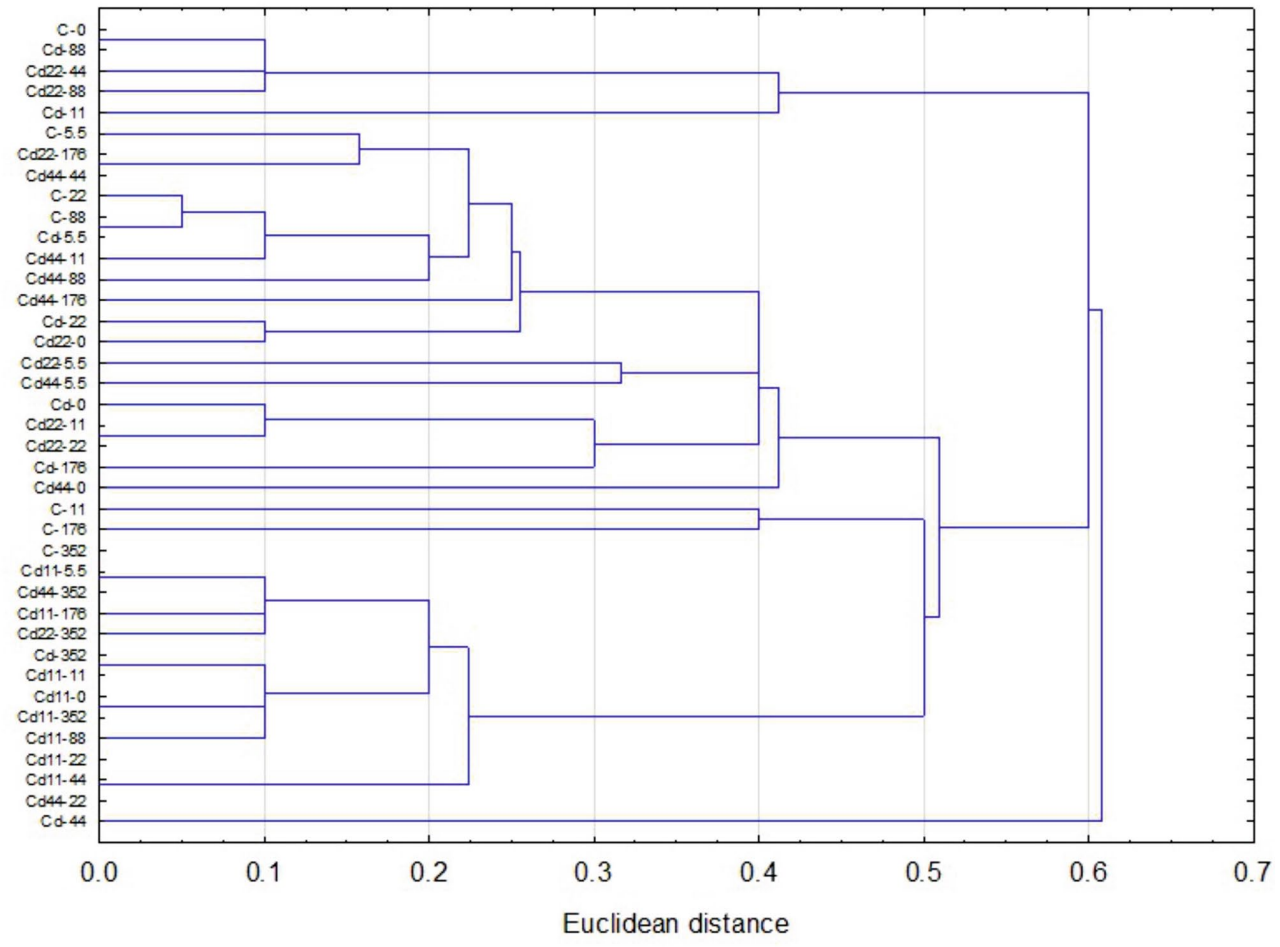
Euclidean distance clustering tree describing the relationships among autophagy parameter (AIR and MAI) values of hemocytes of the 5th larval stage of the moth *S. exigua* from the 2D experiment. Symbols: strain-experimental group.

In general, the highest values of both autophagy parameters were recorded in the groups exposed to the highest Cd concentration. This is clear for each of the strains analyzed; however, the statistical significance of the difference between the values measured for group with the strongest Cd exposure and the remaining experimental groups was confirmed in the case of the strains Cd, Cd22 and Cd44. Regarding strain-related differences, significantly lower expression of autophagy markers is characteristic, in general, for the Cd strain as well as the Cd22 and Cd44 strains. Only among the groups maintained on the highest Cd concentration, for all the strains, were the autophagy parameters equally high (Figs. 1, 2, 3). The control and especially the Cd11 strains demonstrated higher autophagy intensity, separating the experimental groups within this strain from the remaining experimental groups (Fig. 4).

### Midgut

**Figure 5:**
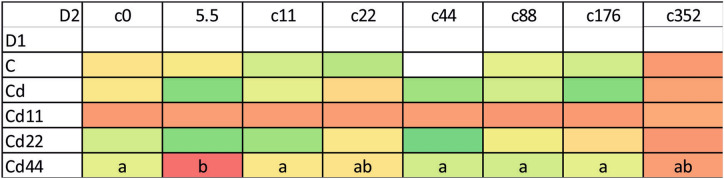
AIR in the midgut of the 5th larval stage of the moth *S. exigua* from the 2D experiment. D1, D2—dimensions of the experiment, according to Fig. 10. Values of the AIR are expressed according to a color scale, where dark green is the lowest and dark red is the highest. Different letters (a, b) denote statistically significant differences between experimental groups (D2); ANOVA, Tukey’s test for unequal sample size, p ≤ 0.05.

**Figure 6:**
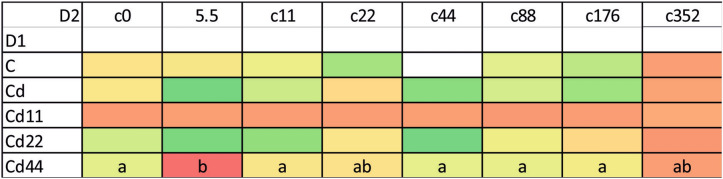
MAI in the midgut of the 5th larval stage of the moth *S. exigua* from the 2D experiment. D1, D2—dimensions of the experiment, according to Fig. 10. Values of the MAI are expressed according to a color scale, where dark green is the lowest and dark red is the highest. Different letters (a, b) denote statistically significant differences between experimental groups (D2); ANOVA, Tukey’s test for unequal sample size, p ≤ 0.05.

**Figure 7:**
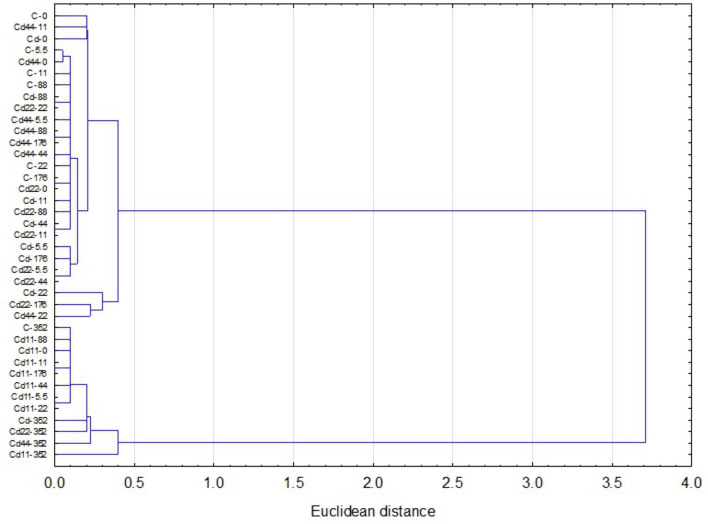
Euclidean distance clustering tree describing the relationships among autophagy parameter (AIR and MAI) values of midgut cells of the 5th larval stage of the moth *S. exigua* from the 2D experiment. Symbols: strain-experimental group.

In the midgut cells, the only significant differences were observed between the values of the Cd5.5 experimental group and the Cd44 strain, which were significantly different from the remaining groups (Figs. 1, 5, 6, 7).

### Life history parameters

**Figure 8:**
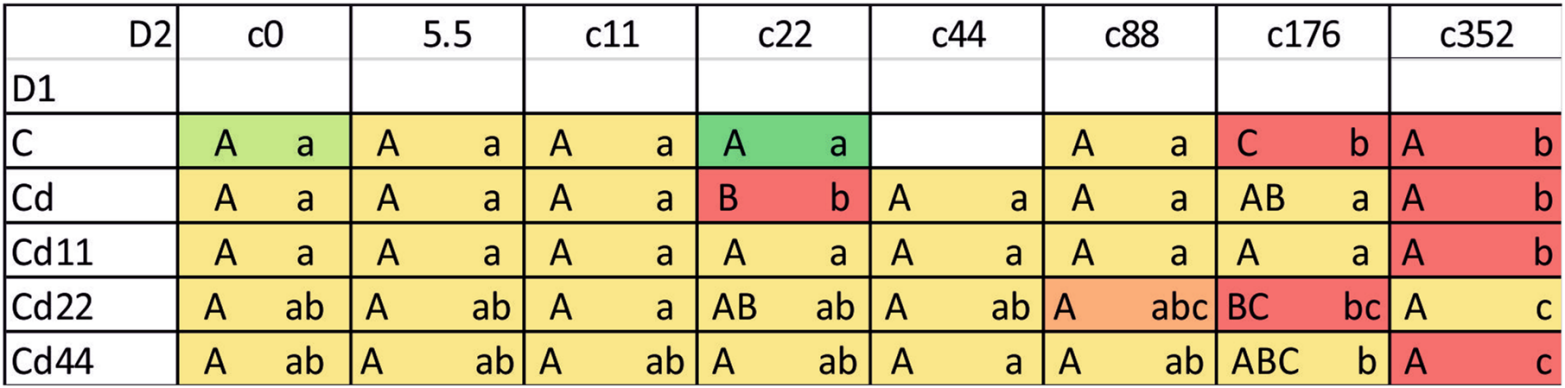
Duration of the 3rd larval instar of the moth *S. exigua* from the 2D experiment. D1, D2—dimensions of the experiment, according to Fig. 10. Values are expressed according to a color scale, where dark green is the lowest and dark red is the highest. Capital letters (A, B, C) denote statistically significant differences between strains (D1), and small letters (a, b, c) denote statistically significant differences between experimental groups (D2); ANOVA, Tukey’s test for unequal sample size, p ≤ 0.05.

**Figure 9:**
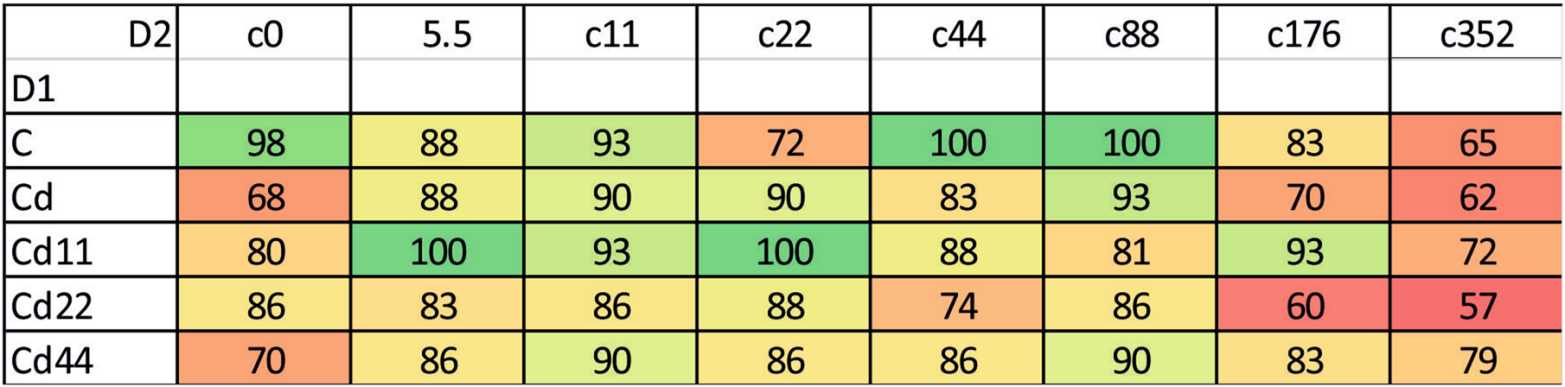
Cumulative mortality of 3rd and 4th larval instars of the moth *S. exigua* from the 2D experiment. D1, D2—dimensions of the experiment, according to Fig. 10. Values are expressed as the % of living individuals and according to a color scale, where dark green is the lowest and dark red is the highest mortality.

Although in all the strains, a significantly higher value was found for the groups exposed to the highest Cd concentration in food, the pattern observed for the duration of the 3rd larval instar appeared to be both strain and group dependent. This parameter was significantly different only if the insects of the strains were exposed to 22 or 176 µg Cd per mg of food (dry mass). In the former case, larvae from the Cd22 strain, and in the latter case, control larvae needed the longest time to complete the 3rd larval stage. In the D2 dimension, for the control strain, in general, the duration of the 3rd larval instar stage reflected the increase in Cd concentration in the food in subsequent experimental groups, with the highest values in the groups exposed to 176 and 352 μg Cd per g of food (dry weight). The duration of the 3rd larval instar stage in those two groups was significantly higher than that in the remaining groups. Similarly, in the Cd strain, the longest duration of the 3rd larval instar stage was also found for the individuals exposed to 22 μg Cd per g of food (dry weight). In the strains exposed to various Cd concentrations for 20 generations, a distinct difference between the highest Cd exposure groups and the remaining groups was found for the Cd11 and Cd44 strains. In the Cd22 group, the duration of the 3rd larval instar stage was homogeneous, with the values counted for the groups fed diets containing 88 and 176 μg Cd per g of food (dry weight) (Fig. 8). Cumulative mortality calculated from the beginning of the 3rd to the end of the 4th instar stage also showed a tendency similar to that of the remaining parameters: for all strains, the highest value was found in the groups exposed to the highest Cd concentration in food. Additionally, in the control groups for the Cd-exposed groups, mortality was relatively high but still lower than that in the groups with the highest Cd intoxication (Fig. 9).

### Correlation analysis

After analyzing each strain separately, both autophagy parameters in the midgut cells were significantly positively correlated with the concentration of cadmium in the food of the insects from experimental groups within the C, Cd and Cd22 strains. In the Cd11 group, a significant negative correlation was found, while in the Cd44 group, no significant correlation was found. In the hemocytes, a significant positive correlation between autophagy markers and Cd concentration in the experimental groups was found in all the strains except the Cd11 strain. For the insects from this strain, no significant correlation was found (Table 2). The duration of the 3rd larval stage of the insects was significantly positively correlated with the Cd concentration in the experimental groups (Table 3). The correlation coefficient describing the relationship between cumulative mortality and Cd concentration in food in the experimental groups was significant only in the Cd22 strain (r = −0.874640, r^2^ = 0.764995, p = 0.004474). The positive correlation between the autophagy parameters and the duration of the 3rd larval stage was significant only in the case of the Cd (gut and hemolymph, positive) and Cd44 strains (gut, positive), and the Cd11 strain showed a negative correlation (gut, negative; Table 4.).

**Table 3:** Pearson’s correlation coefficient describing the relationships between autophagy markers (AIR and MAI) or duration of the 3rd larval instar stage and Cd concentrations in the food of the insects from the experimental groups within each strain.

Strain	Haemolymph		Midgut		Duration of the 3rd larval instar
	AIR	MAI	AIR	MAI	
C	*0.643428*	*0.621136*	*0.860362*	*0.818146*	*0.506164*
Cd	*0.473036*	*0.465378*	*0.681843*	*0.733374*	*0.282352*
Cd11	0.134197	0.014597	−*0.380284*	−*0.524433*	*0.509908*
Cd22	*0.577966*	*0.581286*	*0.857618*	*0.868465*	*0.307634*
Cd44	*0.389825*	*0.391936*	0.097768	0.096842	*0.368861*

Values in italics denote coefficient values significant at p ≤ 0.05.

**Table 4:** Pearson’s correlation coefficient describing the relationships between autophagy markers (AIR and MAI) and duration of the 3rd larval instar stage of the insects from the experimental groups within each strain.

Strain	Haemolymph		Midgut	
	AIR	MAI	AIR	MAI
C	0.713245	0.713076	0.522214	0.512291
Cd	*0.822076*	*0.794996*	*0.855574*	*0.840366*
Cd11	−0.157622	0.218218	−*0.927426*	−*1.000000*
Cd22	0.014106	0.046374	0.040681	0.145173
Cd44	0.635258	0.668153	*0.969727*	*0.965176*

Values in italics denote coefficient values significant at p ≤ 0.05.

## Discussion

The experimental setup presented in this report was established 3 years ago as a basis for the investigation of whether multigenerational exposure to a selection factor that results in increased tolerance to this factor may be the effect of adaptation or may result from phenotypic plasticity. The idea of this 3-year project was presented elsewhere^29^. The experiment presented here covers 160 generations for the main strains (C and Cd) and 20 generations for the remaining strains established for the project (Cd11, Cd22 and Cd44). Therefore, as far as possible, the results will be compared to the results obtained for these exact strains at other time points. It must be emphasized here that to the best of our knowledge, this population of insects with a long-lasting selection history under controlled conditions (Cd strain) is unique worldwide and is still ongoing. The Cd concentrations used for the project are environmentally verified. The initial Cd-selected strain (strain Cd) was exposed to a moderate Cd concentration, which is recognized as sublethal and allows successful reproduction but stimulates defensive processes. Tarnawska et al.^29^ compared the stress protein levels in the C and Cd strains after more than 150 generations and the Cd44 strain in the 1st generation of contact with cadmium at the same concentration administered to the Cd strain. Similar results were also obtained regarding DNA damage levels^31^ and apoptosis (in preparation). A concentration of 44 μg of Cd per g of food (dry weight) is comparable with the cadmium concentration of polluted soils. The Cd concentration in soil treated as polluted ranges from 51 to 82 μg/g^32,33^. Under such conditions, the beetles inhabiting the area are more susceptible to additional stressors. Additionally, Osman et al.^34^ demonstrated that a concentration of 59 μg of Cd per g of soil (dry weight) (vs control: 4.80 μg of Cd/g of soil (dry weight)) enabled a population of the beetle *Blaps polycresta* to exist at a lower density and at the cost of body size and tissue malformations. On the other hand, Nahmani and Rossi^35^ described an area with 190 ppm Cd as highly polluted. Therefore, the concentration of 352 μg of Cd per g of food (dry weight) that was applied in this project can be regarded as high or very high (compare also^36,37^.

**Figure 10:**
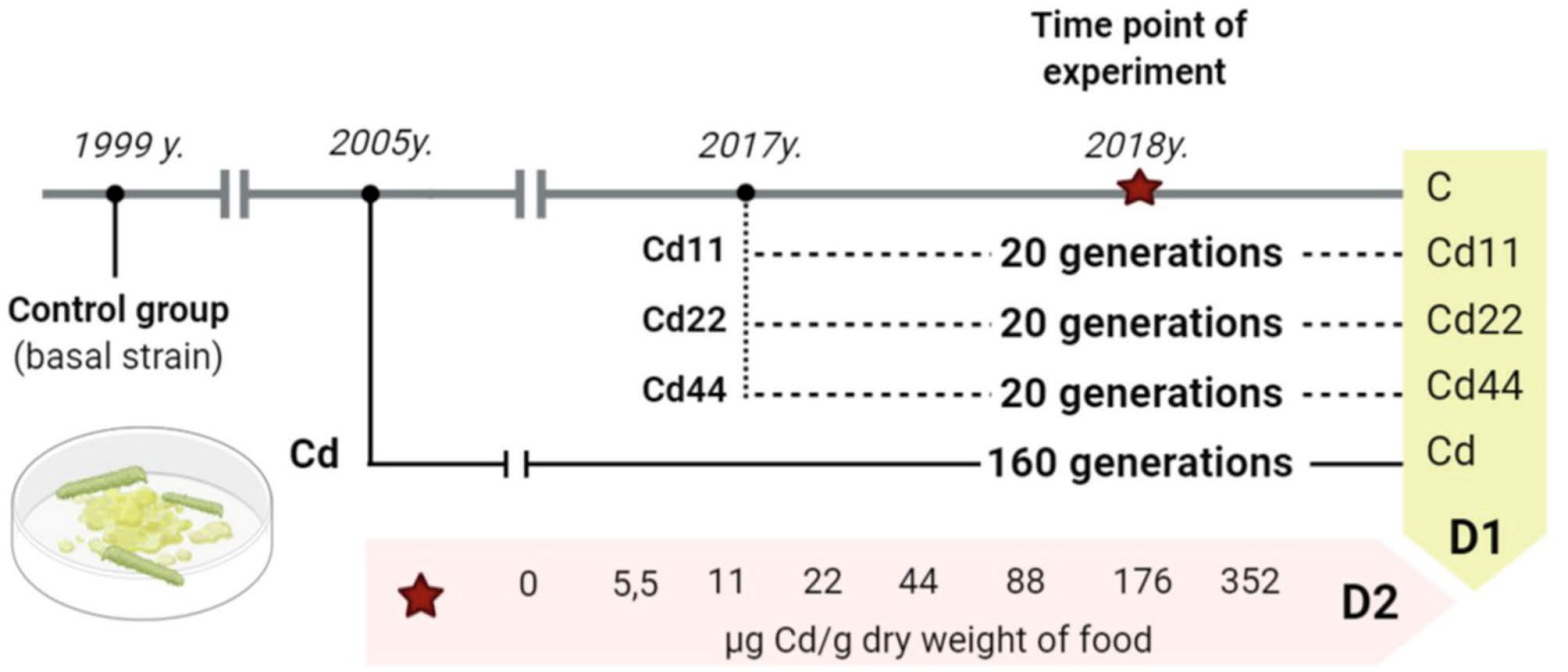
Scheme of the 2D experiment. Asterisks connect the timepoint of the experiment and the experimental groups. D1—strains, D2—experimental groups.

The analysis of the results indicated that the values of autophagy indices were related to both the strain (the number of generations with pre-exposure to cadmium and the concentration of Cd in the diet of the pre-exposed strains, D1, Fig. 10) and the experimental group (with exposure to different Cd cadmium concentrations for a period of ontogenesis, D2, Fig. 10). The statistical homogeneity of the C and Cd strains once again confirms that, also at the level of autophagy processes, the Cd strain has developed relative resistance to the harmful effects of this metal. This is especially visible in the groups reared on Cd-free food, in which the autophagy markers had the lowest values. Similar observations were made in our other experiment. Tarnawska et al.^29^ demonstrated that within the period of 18 generations (146–164 generations of the Cd-selected strain), the contents of stress proteins (HSP70 and Mts) in the Cd strain did not differ from those in the control strain. Therefore, also in this report, we may suppose that in the cells of Cd strain insects, similar to those of the control strain insects, autophagy processes are not initiated early, as observed under the impact of extremely high acute Cd exposure. Between-strain comparisons of autophagy parameters (D1), however, revealed interesting differences between the two groups of strains with respect to the metal concentration in the food of the insects: between the “old” strains (i.e., the ones exposed to cadmium for 160 generations (Cd and C) and the “new” strains (exposed to Cd for 20 generations). Overall, in all the experimental groups, the Cd22 and Cd44 strains do not differ from the 160-generation strains with respect to autophagy parameters, while the values of the markers obtained for the Cd11 strain are significantly higher (Figs. 1, 2, 3, 4, 5, 6). This interesting relationship may be explained in two ways. First, the relative homogeneity of the strains subjected to longer multigenerational Cd concentration exposure may be connected with increasingly efficient selection of individuals for cadmium tolerance. Thus, the individuals from the Cd22 and Cd44 strains subjected to Cd pressure probably do not develop oxidative stress that stimulates very costly autophagy processes. This may mean that potential free radical generation is neutralized by efficient scavenging mechanisms, including, e.g., metallothioneins. In our previous experiment within the same project, we found that the efficiency of selection (understood as a shorter time needed for a biomarker to have a value similar to the reference level) increases with the Cd concentration. In the quoted study^29^, the stress protein level in Cd44 after 18 generations of exposure was similar as in the C and Cd strains. We concluded that this number of generations was sufficient to obtain a population of insects that do not produce increased amounts of Mts and HSP70. Unlike the Cd44 insects, individuals from the Cd22 strains still had higher levels of these proteins. These levels might have been connected to either (or both) free radical elimination or (and) binding of cadmium to the molecules. Here, in the autophagy experiment conducted several generations later, the situation had changed so that Cd22 insects seemed more tolerant to this metal. However, the defense mechanisms of the individuals of the Cd11 strain may still be elevated because cadmium at concentration of 11 μg of Cd per g of food (dry weight) might not have been sufficient to induce tolerance after 20 generations of exposure. On the other hand, these high values of autophagy indices may be an example of the hormesis effect; a comparison revealed that cumulative survivorship was higher among caterpillars of the Cd11 strain than among caterpillars of the remaining strains. This specific moment of selection (generation + Cd concentration) seems to be when the hormesis effect is manifested as intensified autophagy processes and, possibly as a result of these processes, an unaltered development rate. A similar conclusion was drawn from an experiment on the development of blowflies (pupation success) in the presence of several Cd concentrations in food^38^. The authors observed enhanced pupation success at low concentrations of cadmium and reduced pupation success at higher concentrations, which was explained by the hormesis effect. This effect, however, must have its basis at levels of biological organization lower than the organismal level, e.g., cellular, biochemical or molecular. This assumption was confirmed by the studies of Liu et al.^39^, who tested the effects of a series of sublethal Cd concentrations on the polychaete *Perinereis aibuhitensis* in water. As markers of the effects, enzymatic activity (superoxide dismutase, S-transferases, catalase, and acetylcholinesterase) and gene expression levels (*SOD*, *CAT*, heat shock protein 70 (*HSP 70*), heat shock protein 90 (*HSP 90*), cytochrome P450 (*CYP450*), cytochrome oxidase subunit I (*COI*), metalloprotein (*MP*) and vitellogenin (*VTG*)) were analyzed. The authors concluded, based on the typical or inverted U-shaped pattern of changes, that this phenomenon was a manifestation of the hormesis effect. In this case, however, no life history parameters were measured to assess possible benefits for the animals^39^. The analysis of the relationship between life history parameters (final body weight, feed efficiency, weight gain and specific growth rate) and markers of toxicity, such as metallothionein or lipid peroxidation product levels, as well as hepatopancreas histological damage in response to a sublethal Cd concentration (30 mg/kg Cd of the diet), was reported in Ref.^40^. However, any of the markers could explain the enhanced growth parameters, which was clearly emphasized by the authors. In the present study, the relatively unchanged (or not decreased according to expectations based on the comparisons between Cd11 and other strains) growth and survivorship is connected to increased autophagy intensity. Here, the question of the trade-off should be reconsidered. Previously quoted authors also found that the hormetic response in the earlier developmental stage (pupa) may be followed by an increased risk of death in the next stage (adult emergence^38^). This may be explained by limited energy resources. If these resources are allocated to a larger extent to efficient pupation, then insufficient resources are allocated to another process, since pupae are not able to replenish them. This may be the cost of the hormesis effect. The costs may explain the lack of this effect in the other Cd-selected strains that can be regarded as adapted. Thus, hormesis cannot be treated as adaptation but as the costly fight for survival. Therefore, if we follow the assumption that adaptation means saving energy, the Cd11 strain is still probably in the initial, if any, stage of adaptation to this metal. This may suggest that hypothesis H2 may be true. In the other strains subjected to intoxication with higher Cd concentrations (Cd22 and Cd44), there is probably a trade-off between growth/survivorship and protection, and energy is allocated to protective processes, which may also protect the cells against enhanced autophagy.

In the D2 dimension, comparisons were made within groups of insects with the same pre-exposure history, introducing short-term exposure to the Cd concentration gradient in relation to the autophagy indices. In general, in all the strains, a significant increase in autophagy parameters was observed in the groups exposed to the highest Cd concentration in food. This increase was especially visible in the groups exposed to 5.5, 11, 22 and, especially, 176 μg of Cd per g of food (dry weight). In the latter case, it seems that animals exposed to 11 μg of Cd per g of food (dry weight) for 20 generations (Cd11) had defense processes efficient enough to ensure an appropriate course of development. These defense processes also include autophagy, which may be expected to occur as a necessary defense when the less costly defenses appear insufficient. It is noticeable that this increase corresponds to the diminished cumulative survivorship among the caterpillars from the strains from the highest Cd exposure groups as well as the significantly increased duration of the 3rd larval stage. Literature directly linking autophagy to living in habitats with a gradient of environmental pressure is scarce, except for strictly medical surveys on human diseases. Indirectly, as one of the links between metal pollution and autophagy is oxidative stress, we can suppose that if antioxidative defense is impaired, then autophagy can appear as the next possible step of defense. This suggestion can be found in a report from Islam et al.^41^, who found that significantly (twofold or greater) elevated levels of antioxidative parameters (superoxide dismutase and catalase activities), accompanied by a significantly prolonged duration of developmental stages and a decrease in body weight, correlated with Cd concentrations in the body of the muga silkworm *Antheraea assamensis*. The authors indicated that the late effect of metal intoxication may be cell death but did not measure this themselves. In light of the present results, it can be supposed that the increased levels of autophagy markers may be the effect of insufficient—or impaired—antioxidative mechanisms at this Cd concentration. Autophagy can also play a protective role in contexts other than when antioxidative defenses are too weak. It can be activated when numerous external factors influence or penetrate the organ's cells, disrupting the cell membrane barrier^5,7,42^. Autophagy is a survival factor for animal tissues against pathogen infection. When the intercellular junctions between cells are not a sufficient barrier for the entry of microorganisms into cells, these organisms are enclosed and neutralized inside autophagosomes^43,44^. Numerous toxic substances (e.g., heavy metals) accumulate in the cytoplasm in the form of spherocystals/spherites^45,46^. However, when too many spherites accumulate, autophagy enables the discharge of excess toxic substances and protects cells from inflammation^44,47^.

On the other hand, we should analyze the autophagy process not only as a disruption of earlier lines of defense but also as a strategy for conserving energy resources to maintain life processes. This conclusion was drawn by Falfushynska et al.^48^, who found increased levels of autophagy markers (cathepsin D) together with decreased protein concentrations in mussels from the cooling ponds of thermal power plants, which were overcoming strong thermal and pollution stress. The authors explained this increase—and thus autophagy activation—based on the need to meet energy demands^48^.

From the defense and compensation point of view, it seems that the ability to induce autophagy increases the chance of survival in fluctuating environments. This was suggested by Moore et al.^49^ in their very interesting report on marine mussels (one of the extremely rare reports on autophagy in an environmental pollution gradient). The authors found that lysosomal stability had a U shape along the gradient, and the animals at the most polluted site demonstrated a pattern of lysosomal changes similar to that in the control area. It is worth stating here that field studies use animals that exist in their habitat for numerous generations, even if their number is unknown. The authors hypothesize that this upregulation of autophagy may have a selective meaning^49^. Moreover, considering the present study and other results from the project on multigenerational *S. exigua* strains, we can conclude that this process may be the tool or basis for increased tolerance, possibly at the level of phenotypic plasticity. This question was previously discussed in detail^29^.

Last but not least, it is noticeable that the pattern of autophagy indices was nearly uniform in both tissues used for this study; however, the differences were more distinct and significant in hemocytes than in gut cells. This corresponds to the recommendation of Picot et al.^50^, who verified the usefulness of hemocytes of the oyster *Crassostrea gigas* under induction and inhibition conditions. They also explained this result by the fact that hemocytes play a crucial role in animal immunity and defense against pathogens. Additionally, for the same reasons, hemocytes were used as markers of the condition of spiders exposed to cadmium^51^. Moreover, in spiders, hemocytes appeared to be more sensitive to cadmium toxicity than midgut cells, based on the measurement of DNA damage levels^52^. This may also be a good reason to investigate environment-induced autophagy in invertebrates.

In summary, considering all the aspects discussed above, i.e., the energy deficit, oxidative stress effects and the data indicating that autophagy induced by environmental stressors is enhanced at the highest intensity of pressure and diminishes after efficient selection, this process is an efficient but energetically expensive mechanism of defense. Thus, under stress conditions, autophagy represents the ultimate—or penultimate—weapon for preserving and ensuring appropriate biological processes; thus, the H2 hypothesis can be accepted.

## Material and methods

### Insects

*Spodoptera exigua* is a moth species known as an important oligophage pest that infests various kinds of crop plants, including vegetables and cereals. Uncontrolled caterpillars may cause defoliation of the plants. For scientific research, *S. exigua* is often used as a model insect^53,54^. At the Institute of Biology, Biotechnology and Environmental Protection, it has been reared under controlled laboratory conditions since 1999 (control strain, C). In 2005, based on the insects from the initial strain, a new strain was established. This strain was fed with a medium containing 44 μg of Cd per g of food (dry weight) as a selection factor (Cd-intoxicated strain, Cd). Since then, approximately 160 generations of Cd-selected insects have occurred. A detailed description of the rearing methodology can be found in Refs.^23–25^. Moreover, in 2017, from the basal strain, three additional populations were generated, which differed in terms of the Cd concentration in the food (11, 22 and 44 μg of Cd per g of food (dry weight))^29^. Prior to the present experiment, approximately 20 generations of Cd-selected insects had occurred.

### Experimental setup

To establish a 2D experimental setup, the representatives of each strain that differed in terms of Cd concentration and exposure duration (1st dimension) were exposed to one of the increasing Cd concentrations in food for 1 generation (2nd dimension; Fig. 10). Insects from each strain were randomly selected for the experimental groups.

### Sample preparation

The measurements were conducted using the midgut and hemolymph of the 5th larval stage of the *S. exigua* caterpillars. Before the dissection, the individuals were anaesthetized on ice. For hemolymph samples, a proleg of a caterpillar was cut off, and 50 µl of the flowing hemolymph was collected using a pipette. Then, the hemolymph was mixed with 50 µl of anticoagulant buffer (0.14 M NaCl, 0.1 M glucose, 30 mM trisodium citrate, 26 mM citric acid, 10 mM EDTA, pH 4.6 (2:1, v/v)). Samples prepared in this way were regarded as cell suspensions ready for flow cytometry analyses. The same caterpillar was then used to dissect the midgut, which, after cleaning it from the undigested food, was placed into 250 µl of PBS buffer. The gut samples were then shaken in a homogenizer (Minilys, Bertin) to obtain cell suspensions. Each measurement was conducted in 5 replicates.

### Autophagy indices

Autophagy was assessed using two parameters: mean autophagy intensity and autophagy induction ratio, obtained by the flow cytometry method, using a Muse Cell Analyzer (EMD Millipore Corporation) flow cytometer and a Muse Autophagy LC3-Antibody Based Kit, according to the manufacturer’s protocol.

### Life history parameter testing

For each experimental group, 40 one-day-old larvae in the 3rd instar stage were randomly selected, and 10 individuals were placed in a Petri dish (i.e., 4 Petri dishes were assigned for each group; according to the Fig. 10). In total, 400 individuals were included in the experiment. During the experiment, the cumulative survivorship of individuals (3rd and 4th larval stage, i.e., beginning with the 2nd day of the 3rd instar stage and ending with the last day of the 4th instar stage) and the duration of the 3rd instar stage were calculated. Then, 5th instar larvae were used for autophagy analyses.

### Statistical analyses

Data analyses were based on statistical methods. Analyses were conducted using TIBCO Software Inc. (2017) (STATISTICA data analysis software system). ANOVA and Tukey's t test for unequal sample size were applied for post hoc analysis (p < 0.05) of comparisons. To assess the relations between data, clustering-based data aggregation techniques were applied (Euclidean distance tree). Correlation matrices describing relationships between autophagy parameters, the duration of the 3rd larval stage or cumulative mortality and Cd concentration in the experimental groups were established using r-Pearson correlation.

## Supplementary information

Supplementary Information

Supplementary Table S1

Supplementary Table S2
