# Supplementary material for: Autophagy: a necessary defense against extreme cadmium intoxication in a multigenerational 2D experiment
Source: Sci Rep. 2020 Dec 3;10:21141. doi: 10.1038/s41598-020-78316-z (PMC7712871; doi:10.1038/s41598-020-78316-z)
Supplement: Supplementary file 1 — Supplementary Information [file 41598_2020_78316_MOESM1_ESM.docx]

**Autophagy – a necessary defense against extreme cadmium intoxication in a multigenerational 2D experiment**

Agnieszka Babczyńska, Agnieszka Nowak, Alina Kafel, Bartosz Łozowski, Magdalena Rost-Roszkowska, Monika Tarnawska, Maria Augustyniak, Marta Sawadro, Agnieszka Molenda

Institute of Biology, Biotechnology and Environmental Protection, Faculty of Natural Sciences, University of Silesia in Katowice, Bankowa 9, 40-007 Katowice, Poland

Corresponding author: [agnieszka.babczynska@us.edu.pl](mailto:agnieszka.babczynska@us.edu.pl)

Agnieszka Nowak, 226aga@wp.pl

Alina Kafel, alina.kafel@us.edu.pl

Bartosz Łozowski, bartosz.lozowski@us.edu.pl

Magdalena Rost-Roszkowska, magdalena.rost-roszkowska@us.edu.pl

Monika Tarnawska, monika.tarnawska@us.edu.pl

Maria Augustyniak, maria.augustyniak@us.edu.pl

Marta Sawadro, marta.sawadro@us.edu.pl

Agnieszka Molenda; amolenda@us.edu.pl

**LC3 in invertebrates – literature review and BLAST comparisons**

To justify the application of Muse Autophagy LC3-Antibody Based Kit most likely dedicated to human LC3 protein, we have (i) reviewed literature data concerning the assessment of autophagy intensity using immunodetection techniques in invertebrate species (Table S1) and (ii) analyzed the identity of human (mouse) LC3 protein sequence with available invertebrate LC3 sequences using alignment tool (BLAST).

Table S1. Results of literature research

| species | Antibody | Species reactivity* | reference |
| --- | --- | --- | --- |
| **sea urchins**, *Strongylocentrotus intermedius*  **mussel** *Mytilus galloprovincialis* | Anti-LC3 antibody produced in rabbit | mouse, rat, human | [1,4] |
| **cricket** *Troglophilus neglectus* harvestmen *Gyas annulatus* | LC3B primary antibody | Human, mouse, rat | [2,3] |
| crab, *Eriocheir sinensis*prawn Macrobrachium rosenbergii | anti-LC3B | rat, human, mouse | [5,6] |

*according to the antibody manufacturer’s information

(i). The example results of the research are given in the table. The authors use primary antibodies that are commercially available. Acccording to the manufacturers of each antibody the papers refer to, their species reactivity include mouse, rat and human in all reviewed cases. This is acceptable, since the autophagy steering mechanism is highly conserved [7, 8].

(ii). To make the comparisons, we have aligned LC3 protein sentences for available invertebrate data (3 invertebtare species sequences available in ncbi database: the sea cucumber, *Apostichopus japonicus,* the shrimp, *Penaeus vannamei;* and the crayfish, [*Cherax quadricarinatus*](https://www.ncbi.nlm.nih.gov/protein/QBQ52881.1)). The results are given in the set of tables, below. Since the manufacturers guarantee species reactivity for human, rats and mouse, the first comparison was done for human and mouse pair of sequences. Then, having obtained 100% identity, the remaining comparisons were conducted just between human sequence and individual invertebrate sequence. Also, since in the references 1 and 4 the authors did not refer to the LC3 isoform, while the authors of references 2, 3, 5 and 6 did, we have included both isoforms into the analyses. The papers 1, 5 and 6 refer to the animals closely related to the species, for which the LC3 sequence are available in the ncbi database. Terrestrial invertebrates (namely insects or arachnids are not represented in the database. However, they have been successfully studied using vertebrate-dedicated antibodies [2,3]), similarly as mussels [4] basing on high conservativeness of the autophagy pathways at the levels of genes and proteins.

Concluding, literature review give us arguments for using the Muse® Autophagy LC3-Antibody Based Kit for the present study. Especially if our aim is not to mechanistically describe the autophagy mechanisms themselves but to put the process in a wider context of adaptaive or microevolutionary context.

1. Kalachev, A.V., Yurchenko, O.V. & Kiselev K.V. Macroautophagy is involved in residual bodies formation during spermatogenesis in sea urchins, Strongylocentrotus intermedius. *Tissue and Cell* **56**, 79-82 (2019).

2. Lipovšek, S. & Novak, T. Autophagy in the fat body cells of the cave cricket Troglophilus neglectus Krauss, 1878 (Rhaphidophoridae, Saltatoria) during overwintering. *Protoplasma* **253**, 457–466 (2016).

3. Lipovšek, S., Janžekovič, F. & Novak, T. Autophagic activity in the midgut gland of the overwintering harvestmen Gyas annulatus (Phalangiidae, Opiliones). *Arthropod Struct. Dev.* **43(5)**, 493-500 (2014).

4. Balbi, T. *et al*. Autophagic processes in *Mytilus galloprovincialis* hemocytes: Effects of *Vibrio tapetis***.** *Fish Shellfish Immunol.* **73**, 66-74 (2018).

5. Sun, M. *et al*. CpG ODNs induced autophagy via reactive oxygen species (ROS) in Chinese mitten crab, *Eriocheir sinensis*. *Dev. Comp. Immunol.* **52(1)**, 1-9 (2015).

6. Sirorat, T. et al. Autophagy-associated shrinkage of the hepatopancreas in fasting male *Macrobrachium rosenbergii* is rescued by neuropeptide F. *Front. Physiol.* **9**, 613 (2018).

7. Chera, S., Buzgariu, W., Ghila, L. & Galliot, B. Autophagy in hydra: A response to starvation and stress in early animal evolution. Biochimica et Biophysica Acta (BBA). *Mol. Cell Res.* **1793(9)**, 1432-1443 (2009).

8. Levine, B. & Klionsky, D.J. Development by Self-Digestion: Molecular mechanisms and biological functions of autophagy*. Dev. Cell*. **6(4)**, 463-477 (2004).

Human (isoform a) vs mouse:

| max score | total score | query cover | E value | Per. Ident. | accession |
| --- | --- | --- | --- | --- | --- |
| 250 | 250 | 100% | 7e-93 | 100.00% | Query_19641 |

Human (isoform a) vs *Apostichopus japonicus*

| max score | total score | query cover | E value | Per. Ident. | accession |
| --- | --- | --- | --- | --- | --- |
| 160 | 160 | 97% | 6e-57 | 61.02% | Query_62141 |

Human (isoform a) vs *Penaeus vannamei*

| max score | total score | query cover | E value | Per. Ident. | accession |
| --- | --- | --- | --- | --- | --- |
| 182 | 182 | 95% | 6e-66 | 70.69% | Query_5253 |

Human (isoform a) vs [*Cherax quadricarinatus*](https://www.ncbi.nlm.nih.gov/protein/QBQ52881.1)

| max score | total score | query cover | E value | Per. Ident. | accession |
| --- | --- | --- | --- | --- | --- |
| 134 | 134 | 90% | 4e-47 | 55.45% | Query_42851 |

Human (isoform b) vs mouse:

| max score | total score | query cover | E value | Per. Ident. | accession |
| --- | --- | --- | --- | --- | --- |
| 223 | 223 | 86% | 3e-82 | 100.00% | Query_51441 |

Human (isoform b) vs *Apostichopus japonicus*

| max score | total score | query cover | E value | Per. Ident. | accession |
| --- | --- | --- | --- | --- | --- |
| 147 | 147 | 85% | 7e-52 | 62.62% | Query_57673 |

Human (isoform b) vs ***Penaeus vannamei***

| max score | total score | query cover | E value | Per. Ident. | accession |
| --- | --- | --- | --- | --- | --- |
| 167 | 167 | 85% | 3e-60 | 71.03% | Query_64187 |

Human (isoform b) vs [*Cherax quadricarinatus*](https://www.ncbi.nlm.nih.gov/protein/QBQ52881.1)

| max score | total score | query cover | E value | Per. Ident. | accession |
| --- | --- | --- | --- | --- | --- |
| 133 | 133 | 85% | 1e-46 | 56.07% | Query_18715 |
