# Supplementary material for: Autophagy: a necessary defense against extreme cadmium intoxication in a multigenerational 2D experiment
Source: Sci Rep. 2020 Dec 3;10:21141. doi: 10.1038/s41598-020-78316-z (PMC7712871; doi:10.1038/s41598-020-78316-z)
Supplement: Supplementary file 2 — Supplementary Table S1 [file 41598_2020_78316_MOESM2_ESM.docx]

Table S1. Mean autophagy intensity and autophagy intensity ratio in the haemocytes and midgut cells of 5^th^ larval instar of the moth *S. exigua* in 2D multigenerational experiment. Mean ± SD.

|  |  | haemocytes | | midgut | | |
| --- | --- | --- | --- | --- | --- | --- |
| \| strain (D1) \| \| --- \| | \| concentration (D2) \| \| --- \| | MAI  mean ± SD | AIR  mean ± SD | MAI  mean ± SD | AIR  mean ± SD | |
| C | 0 | 10.60 | 1,00 | 11,50 | 1,00 | |
|  | 5.5 | 14.65 ± 2.19 | 1,35 ± 0,21 | 10,90 ± 0,99 | 0,95 ± 0,07 | |
|  | 11 | 15.58 ± 0.56 | 1,46 ± 0,05 | 10,68 ± 0,22 | 0,90 ± 0,00 | |
|  | 22 | 13.85 ± 1.14 | 1,30 ± 0,08 | 10,06 ± 0,40 | 0,88 ± 0,04 | |
|  | 44 |  |  |  |  |  |
|  | 88 | 14.08 ± 1.24 | 1,32 ± 0,13 | 10,54 ± 0,47 | 0,92 ± 0,04 | |
|  | 176 | 15.52 ± 0.66 | 1,46 ± 0,05 | 10,24 ± 0,30 | 0,90 ± 0,00 | |
|  | 352 | 17.10 ± 0.48 | 1,62 ± 0,08 | 16,82 ± 1,53 | 1,50 ± 0,00 | |
| Cd | 0 | 12.26 ± 2.02 | 1,18 ± 0,18 | 10,94 ± 0,70 | 0,96 ± 0,05 | |
|  | 5.5 | 12.94 ± 2.38 | 1,20 ± 0,20 | 9,68 ± 0,41 | 0,84 ± 0,05 | |
|  | 11 | 11.66 ± 2.14 | 1,12 ± 0,19 | 10,36 ± 0,74 | 0,92 ±0,04 | |
|  | 22 | 14.94 ± 0.98 | 1,42 ± 0,08 | 12,40 ± 1,36 | 1,10 ± 0,12 | |
|  | 44 | 11.24 ± 2.07 | 1,04 ± 0,22 | 9,86 ± 0,40 | 0,86 ± 0,05 | |
|  | 88 | 10.68 ± 0.64 | 1,02 ± 0,08 | 10,42 ± 0,29 | 0,90 ± 0,00 | |
|  | 176 | 12.28 ± 1.39 | 1,16 ± 0,11 | 10,02 ± 0,63 | 0,84 ± 0,05 | |
|  | 352 | 16.62 ± 0.30 | 1,58 ± 0,04 | 16,58 ± 0,79 | 1,44 ± 0,09 | |
| Cd11 | 0 | 16.54 ± 0.38 | 1,56 ± 0,05 | 17,20 ± 0,58 | 1,50 ± 0,07 | |
|  | 5.5 | 17.14 ± 0.60 | 1,62 ± 0,04 | 16,86 ± 0,32 | 1,46 ± 0,05 | |
|  | 11 | 16.62 ± 0.35 | 1,56 ± 0,05 | 17,04 ± 0,44 | 1,48 ± 0,04 | |
|  | 22 | 16.46 ± 0.53 | 1,54 ± 0,05 | 17,06 ± 0,57 | 1,48 ± 0,04 | |
|  | 44 | 16.50 ± 0.50 | 1,54 ± 0,05 | 16,96 ± 0,38 | 1,46± 0,05 | |
|  | 88 | 16.66 ± 0.68 | 1,58 ± 0,04 | 17,30 ± 0,31 | 1,52± 0,04 | |
|  | 176 | 16.98 ± 0.46 | 1,60 ± 0,00 | 17,06 ± 0,17 | 1,50 ± 0,00 | |
|  | 352 | 16.60 ± 0.46 | 1,58 ± 0.04 | 16,14 ± 0,23 | 1,40 ± 0,00 | |
| Cd22 | 0 | 14.44 ± 1.50 | 1,36 ± 0,17 | 10,38 ± 1,09 | 0,90 ± 0,12 | |
|  | 5.5 | 12.94 ± 2.14 | 1,22 ± 0,18 | 9,74 ± 0,43 | 0,84 ± 0,05 | |
|  | 11 | 12.78 ± 1.86 | 1,22 ± 0,18 | 9,92 ± 0,36 | 0,86 ± 0,05 | |
|  | 22 | 12.02 ± 1.42 | 1,14 ± 0,13 | 11,18 ± 2,25 | 0,96 ± 0,19 | |
|  | 44 | 11.30 ± 1.98 | 1,08 ± 0,19 | 9,66 ± 0,18 | 0,82 ± 0,04 | |
|  | 88 | 11.56 ± 1.61 | 1,10 ± 0,14 | 10,72 ± 1,45 | 0,94 ± 0,11 | |
|  | 176 | 13.90 ± 1.47 | 1,32 ± 0,13 | 12,50 ± 2,10 | 1,08 ± 0,19 | |
|  | 352 | 17.28 ± 0.91 | 1,62 ± 0,08 | 17,60 ± 0,27 | 1,52 ± 0,04 | |
| Cd44 | 0 | 12.62 ± 2.48 | 1,18 ± 0,26 | 10,56 ± 0,67 | 0,92 ± 0,04 | |
|  | 5.5 | 12.76 ± 1.56 | 1,22 ± 0,16 | 20,78 ± 23,32 | 1,80 ± 2,01 | |
|  | 11 | 13.46 ± 1.48 | 1,26 ± 0,15 | 11,10 ± 0,75 | 0,96 ± 0,05 | |
|  | 22 | 16.34 ± 0.27 | 1,54 ± 0,05 | 11,66 ± 0,80 | 1,00 ± 0,07 | |
|  | 44 | 14.52 ± 1.21 | 1,36 ± 0,11 | 10,58 ± 0,27 | 0,90 ± 0,00 | |
|  | 88 | 14.28 ± 2.02 | 1,34 ± 0,21 | 10,62 ± 0,13 | 0,90 ± 0,00 | |
|  | 176 | 13.34 ± 1.26 | 1,28 ± 0,11 | 10,78 ± 0,53 | 0,92 ± 0,04 | |
|  | 352 | 16.56 ± 0.95 | 1,56 ± 0,11 | 16,82 ± 0,73 | 1,46 ± 0,09 | |
