# Supplementary material for: Autophagy: a necessary defense against extreme cadmium intoxication in a multigenerational 2D experiment
Source: Sci Rep. 2020 Dec 3;10:21141. doi: 10.1038/s41598-020-78316-z (PMC7712871; doi:10.1038/s41598-020-78316-z)
Supplement: Supplementary file 3 — Supplementary Table S2 [file 41598_2020_78316_MOESM3_ESM.docx]

Table S2. 3^rd^ instar duration of the moth *S. exigua* in 2D multigenerational experiment. Mean ± SD.

|  |  |  | |
| --- | --- | --- | --- |
| \| strain (D1) \| \| --- \| | \| concentration (D2) \| \| --- \| | 3^rd^ instar duration | |
| C | 0 | 1.70 ± | 0.82 |
|  | 5.5 | 1.76 ± | 0.82 |
|  | 11 | 1.64 ± | 0.71 |
|  | 22 | 1.86 ± | 1.00 |
|  | 44 | 1.98 ± | 0.16 |
|  | 88 | 1.98 ± | 0.94 |
|  | 176 | 3.00 ± | 0.00 |
|  | 352 | 3.09 ± | 1.38 |
| Cd | 0 | 1.91 ± | 0.67 |
|  | 5.5 | 1.90 ± | 0.55 |
|  | 11 | 1.89 ± | 0.76 |
|  | 22 | 3.00 ± | 0.00 |
|  | 44 | 1.71 ± | 0.52 |
|  | 88 | 1.79 ± | 0.70 |
|  | 176 | 2.19 ± | 0.91 |
|  | 352 | 3.00 ± | 1.61 |
| Cd11 | 0 | 1.78 ± | 0.48 |
|  | 5.5 | 1.92 ± | 0.42 |
|  | 11 | 1.98 ± | 0.28 |
|  | 22 | 2.00 ± | 0.00 |
|  | 44 | 2.08 ± | 0.35 |
|  | 88 | 1.95 ± | 0.52 |
|  | 176 | 2.00 ± | 0.00 |
|  | 352 | 2.92 ± | 0.97 |
| Cd22 | 0 | 2.38 ± | 1.29 |
|  | 5.5 | 2.11 ± | 0.88 |
|  | 11 | 1.86 ± | 0.82 |
|  | 22 | 2.26 ± | 0.89 |
|  | 44 | 2.11 ± | 0.95 |
|  | 88 | 2.39 ± | 0.92 |
|  | 176 | 2.97 ± | 1.47 |
|  | 352 | 3.25 ± | 2.03 |
| Cd44 | 0 | 1.91 ± | 0.67 |
|  | 5.5 | 1.90± | 0.55 |
|  | 11 | 1.89 ± | 0.76 |
|  | 22 | 1.79 ± | 0.53 |
|  | 44 | 1.71 ± | 0.52 |
|  | 88 | 1.79 ± | 0.70 |
|  | 176 | 2.30 ± | 0.97 |
|  | 352 | 2.94 ± | 1.66 |
